# A nationwide cross-sectional review of in-hospital hepatitis B virus testing and disease burden estimation in Ghana, 2016 - 2021

**DOI:** 10.1186/s12889-022-14618-3

**Published:** 2022-11-23

**Authors:** Yvonne Ayerki Nartey, Rafiq Okine, Atsu Seake-Kwawu, Georgia Ghartey, Yaw Karikari Asamoah, Kafui Senya, Amoako Duah, Alex Owusu-Ofori, James Amugsi, Damasus Suglo, Sally Afua Bampoh, Lindsey Hiebert, Henry Njuguna, John W. Ward, Amelie Plymoth, Lewis Rowland Roberts, Ansumana Sandy Bockarie, Yaw Asante Awuku, Dorcas Obiri-Yeboah

**Affiliations:** 1 Department of Medical Epidemiology and Biostatistics, Karolinska Institute, Stockholm, Sweden; 2 Department of Internal Medicine, Cape Coast Teaching Hospital, Cape Coast, Ghana; 3 World Health Organisation, Country Office, Accra, Ghana; 4 National Viral Hepatitis Control Program, Ghana Health Service, Accra, Ghana; 5 Ghana Field Epidemiology and Laboratory Training Programme, School of Public Health, University of Ghana, Legon, Ghana; 6 Department of Internal Medicine, University of Ghana Medical Centre, Accra, Ghana; 7 Clinical Microbiology Unit, Komfo-Anokye Teaching Hospital, Kumasi, Ghana; 8 Internal Medicine and Out-patient Department, Sandema District Hospital, Sandema, Ghana; 9 Drive for Health Foundation Ghana, Tamale, Ghana; 10 Department of Internal Medicine, Greater Accra Regional Hospital, Accra, Ghana; 11 Coalition for Global Hepatitis Elimination, Task Force for Global Health, Decatur, GA USA; 12 Department of Medicine, Division of Gastroenterology and Hepatology, Mayo Clinic Rochester, Rochester, MN USA; 13 Department of Internal Medicine, School of Medical Sciences, University of Cape Coast, Cape Coast, Ghana; 14 Department of Medicine, University of Health and Allied Science, Ho, Ghana; 15 Department of Microbiology and Immunology, School of Medical Sciences, University of Cape Coast, Cape Coast, Ghana

**Keywords:** Hepatitis B, Testing, Seroprevalence, Ghana, Sub-Saharan Africa, Viral hepatitis elimination, Children, Mother-to-child-transmission

## Abstract

**Background and aims:**

Data are needed to inform hepatitis B virus (HBV) testing and treatment policies in Ghana to make progress towards achieving the 2030 WHO elimination targets. This study investigated testing patterns for HBV and described the age, sex, and region-specific prevalence of HBV infection in Ghana using hospital data.

**Methods:**

A nationwide multi-centre cross-sectional study was performed where hospital-based registers were reviewed. These included review of 139,966 laboratory, 169,048 blood bank, and 83,920 delivery register entries from 22 healthcare institutions in Ghana. Frequencies and proportions, and crude and pooled estimates reported. Chi squared test was used for tests of independence. Logistic regression was used to identify factors associated with a positive test result.

**Results:**

The crude HBsAg seroprevalence was 8.48% (95%CI 8.25–8.57%) with pooled estimate of 11.40% (95%CI 10.44–12.35). HBsAg seroprevalence among children under 5 years was 1.87% (95%CI 1.07-3.27) and highest age-specific seroprevalence was in those 40-49 years. The highest region-specific seroprevalences was in the Savannah (22.7%). Predictors of a positive HBsAg RDT test included female sex (OR 0.81 95% CI 0.74–0.88), and age (OR 1.005 95%CI 1.002–1.007). The proportion of parturient women receiving HBsAg testing increased between 2017 (87.2%) and 2020 (94.3%) (*p* < 0.001). The crude HBsAg seroprevalence in parturient women was 6.14% (95% CI 5.97-6.31). Among blood donors the crude HBsAg seroprevalence was 5.69% (95%CI 5.58–5.80). Data from 2 teaching hospitals indicated that in 2020, although 1500 HBsAg positive tests were recorded only 746 serological profile and 804 HBV DNA tests were performed. HBV e antigen seroprevalence was 6.28% (95%CI 4.73–7.84).

**Conclusion and recommendations:**

Ghana remains a country with high HBV burden. There is an unequal distribution, with higher HBsAg seroprevalence in the north of the country. Furthermore, PCR testing is not widely available outside of large teaching hospitals, which limits diagnostic work-up. Hepatitis reporting systems and registers should be improved to facilitate data capture of indicators and standardised across the country to allow for comparability. Furthermore, where gains have been made in testing among pregnant women, there is a need for linkage to appropriate care.

## Background

The prevalence of chronic Hepatitis B virus (HBV) infection remains unequally distributed globally, and the highest disease burden is found in southeast Asia and sub-Saharan Africa (SSA) [1, 2]. The high endemicity in these regions is of concern because HBV infection is a significant risk factor for the development of end-stage liver disease, including liver cirrhosis and hepatocellular carcinoma (HCC). It is estimated that globally, over 1.3 million deaths in 2017 were because of liver cirrhosis alone. Studies in West Africa have shown that HBV accounts for 38-59% of cirrhosis and chronic liver disease cases [3–6]. The incidence of HCC continues to increase worldwide, and the highest rates of morbidity and mortality occur in sub-Saharan Africa [7, 8], with HBV infection accounting for 55-70% of HCC cases in the region [3, 9].

To reduce the morbidity and mortality associated with liver cirrhosis and liver cancer, measures to target risk factor reduction, including the elimination of viral hepatitis, especially in highly endemic countries, are of utmost importance. In view of this, the World Health Assembly set out targets for viral hepatitis elimination by 2030 [10]. To achieve these elimination targets, interventions such as effective vaccination of viral hepatitis, prevention of mother-to-child transmission, injection, blood and surgical safety, harm reduction practices in injecting drug users and treatment of viral hepatitis are recommended [11]. There is a need to scale up testing and treatment of HBV infection in sub-Saharan Africa. This in turn requires up-to-date estimates of the disease burden and an accurate picture of the challenges to testing and treatment that exist in countries with high disease burden.

Viral hepatitis poses a significant public health threat in Ghana, and the country is considered to have high endemicity of Hepatitis B infection (i.e., prevalence ≥8%) [12, 13]. In 2019, there were over 3500 deaths due to viral hepatitis with about 50% of liver cancer deaths attributable to HBV and 14% attributable to HCV [14]. The national seroprevalence of HBsAg in a systematic review by Ofori-Asenso and Agyeman in 2016 was reported to be 12.3% [13], whilst a more recent review by Abesig et al. published in 2020 estimated the prevalence at 8.4% in the adult population [12].

There are several limitations in estimating a nationally reflective HBV prevalence from studies in Ghana, including a lack of data from all administrative regions in the country, the use of estimates based on studies mostly conducted in voluntary and replacement blood donors or specific patient groups, and limited data from children and adolescents. Furthermore, following the creation of new administrative regions by the government of Ghana in 2019, in which the number was increased from 10 to 16 [15], a large-scale study which describes the current national burden encompassing these new regions is yet to be published. Consequently, there are still gaps that remain in estimation of the district and consequently regional-level, age and gender specific burden of HBV infection in Ghana.

This study investigated the testing patterns for hepatitis B virus infection in Ghana, estimated the age- gender- and region- specific prevalence of HBV infection. Findings from this study provides information that will guide the formulation of policies and interventions to improve hepatitis B testing and treatment services in Ghana.

## Methods

### Study design

A nationwide cross-sectional study to describe testing patterns and burden of HBV infection using existing hospital-based registers across multiple sites in Ghana.

### Sampling approach

Using purposive sampling, Ghana Health Service affiliated healthcare institutions and HBV related non-governmental organizations (NGO’s) were selected for data collection. Selection was based on zoning of Ghana’s 16 administrative regions into the northern, middle, and southern belt. In each zone, two regional hospitals, 2 district-level hospitals, 1 faith-based facility, 1 HBV-related NGOs, public health reference laboratories (PHRL), and teaching hospitals present in the zone were selected by convenience sampling and approached for data collection. Out of 26 sites approached, 22 (84.6%) provided data.

### Data collection

From February 2021 to December 2021, paper-based laboratory, blood bank and delivery ward register records spanning entries from 1st January 2016 to 1st January 2021 were reviewed across study sites. Field work in each institution took 2 to 4 days. Data reviewed were reported in registers either as monthly or yearly aggregated results, or as results for each person tested on a case-by-case basis. Data included number of positive cases, number of negative cases and total number tested. Much of the data was from paper-based records that were retrieved from archives. In two centres, data were obtained from electronic health information management systems instead of paper-based records. Where available, data on age and gender within the records were obtained.

Information on types of HBV tests available including point of care (POC) tests such as rapid diagnostic tests (RDTs) for HBsAg and HBV serologic profile, and centralized tests such as Enzyme Linked Immunosorbent Assay (ELISA) were collected for each site for the review period (2021). Additionally, we evaluated the crude number of HBsAg, HBV serological profile including HBV core antibody (HBcAb) and HBV envelope antigen (HBeAg), and HBV DNA testing conducted per facility. We performed testing capacity to determine the proportion of sites visited conducting various types of HBV-related tests, and further evaluated cost of testing. From labour ward delivery registers, we collected data on HBsAg testing of pregnant women, and the proportion of pregnant women who had undertaken a test by the time of delivery across all study sites. Data were extracted using a data extraction form designed for data capture, and extracted data was entered into a Microsoft Excel template.

### Statistical analysis

Patient characteristics including age and sex were described using means with standard deviation and median with interquartile range for continuous variables, and frequencies (percentages) for categorical variables. Chi squared test was used to compare proportions in independent groups of categorical variables. We used multivariable logistic regression to identify factors associated with a positive HBsAg test result and adjusted for age (continuous) sex (male, female), year (categorical) and study region (categorical). All tests were two-sided and a *p* value of less than 0.05 was considered statistically significant. Data analysis was performed using Stata, version 17; StataCorp software.

## Results

### Records reviewed and availability of data

**Fig. 1:**
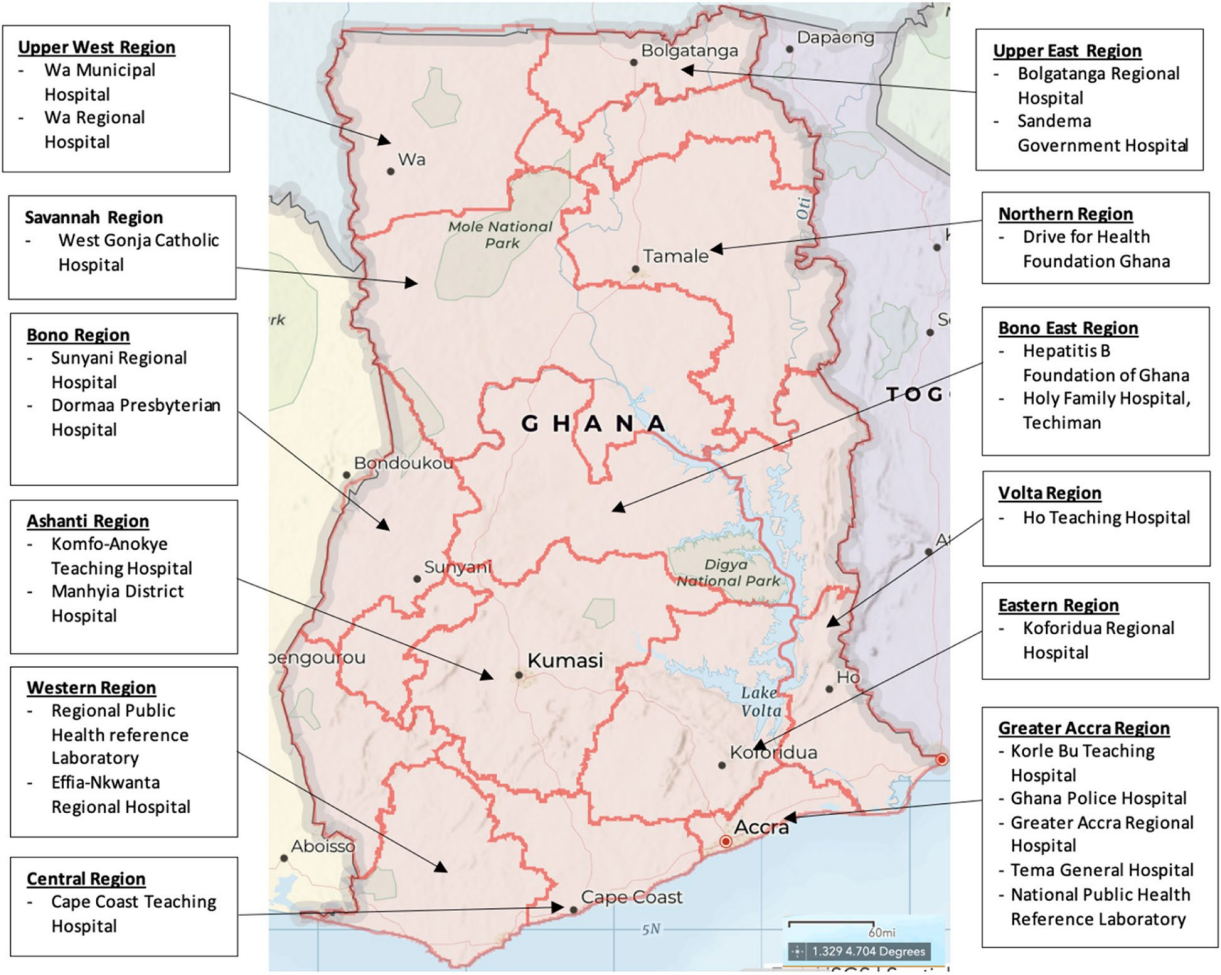
Map of Ghana showing study site locations. Map source: https://arcg.is/15SS4X (ArcGIS hosted by ESRI. Attribution: HERE, Garmin, FAO, NOAA, USGS)

We reviewed a total of 139,966 laboratory register entries comprising 43,609 individual patient data and 96,357 monthly or yearly aggregated records from January 2016 to January 2021 in 22 healthcare institutions. These comprised 4 teaching hospitals, 6 regional hospitals, 4 district hospitals, 3 faith-based institutions, 1 quasi government hospital, 2 public health reference laboratories and 2 NGO’s which spanned 12 out of the 16 administrative regions in Ghana (Fig. 1). The availability of records for the 5-year period under review varied from one study site to another due to damage to or difficulty finding archived records. Consequently, records reviewed varied by year and by type of data between different study sites. All study sites provided data laboratory register data for the year 2020. In laboratory registers, all HBsAg testing was performed using RDTs. The reasons for testing were not captured in registers. However, laboratory personnel reported most tests performed were conducted on account of risk-based assessment on a doctor- or health worker- initiated basis. The brand of test kits used by facilities were not captured in laboratory registers, however it was reported that supply of test kits was based on availability from the Ghana Health Service procurement and supply chain. For NGO’s HBV testing was based on community-based or population-based screening and was conducted periodically when funds were available.

Additional data were retrieved from 83,920 delivery register records and 169,048 blood bank register entries, for review and analysis. For blood bank registers, facilities reported that for each potential blood donor, a pre-screening questionnaire to identify at-risk practices was carried out as part of the Ghana National Blood Transfusion Service protocols. If individuals were identified to have specific risks as per the protocol, they would be disqualified from proceeding with testing, for subsequent blood donation. In blood bank registers reviewed, there was no categorization into voluntary or replacement blood donors because this information was not routinely recorded across all centres.

### Laboratory capacity assessment

Majority of study sites did not perform HBV DNA testing, and we found that only 2 out of 22 sites routinely performed this test (Table 1). These were teaching hospitals located in the Greater Accra and Ashanti Regions. An additional 4 sites had the capacity to perform HBV DNA, however their PCR equipment was non-functional at the time of fieldwork and data collection. In the remaining sites (16 out of 22), patients were sent to private institutions or teaching hospitals for testing to be performed since they lacked the necessary equipment. Data on HBV DNA were therefore only available from two teaching hospitals which routinely performed these tests. Furthermore, of the 14 referral centres outside of teaching hospitals, 12 were not running ELISA based serological profile tests, although RDT kits for HBV serological profile were available in all but one of the sites.

**Table 1:** Testing capacity of health facilities for HBV-related investigations (2021)

	Teaching	Regional	District	CHAG/QUASI	PHRL	NGO	Total n/N (%)
HBsAg	4/4	6/6	4/4	4/4	2/2	2/2	22/22 (100)
HBV Profile (RDT)	4/4	6/6	3/4	4/4	2/2	0/2	19/22 (86.4)
HBV Profile (ELISA)	3/4	2/6	0/4	0/4	1/2		6/20 (30.0)
HBV DNA^a^	4/4	0/6	0/4	1/4	1/2		6/20 (30.0)
Liver Function Test	4/4	6/6	3/4	4/4			17/18 (94.4)
INR	4/4	3/6	0/4	1/4			8/18 (44.4)
Abdominal Ultrasound	4/4	6/6	4/4	4/4			18/18 (100)
Abdominal CT^b^	4/4	2/6	0/4	2/4			8/18 (44.4)
Endoscopy	2/4	2/6	0/4	2/4			6/18 (33.3)
Biopsy	3/4	0/6	0/4	0/4			3/18 (16.7)
Genotyping	0/4	0/6	0/4	0/4			0/18 (0)

*NB:* area shaded in grey represents tests that fall out of the purview of these institutions

*Abbreviations: RDT* Rapid diagnostic test, *ELISA* Enzyme-linked immunosorbent assay, *INR* International normalised ratio

^a^Only two centres (teaching hospitals) had functional PCR machines at the time of assessment

^b^Only two centres (teaching hospitals) had functional CT scans at the time of assessment

### Cost of testing

A summary of the testing costs for HBV-related care is summarized in Table 2. There were differences in testing prices across various facilities, ranging from free or subsidized pricing if one had valid national (government) health insurance, to non-subsidized for those without health insurance. Notably, neither HBV DNA testing nor antiviral therapy with tenofovir were subsidized if a patient had government health insurance, therefore patients were expected to pay for the full cost. Furthermore, for pregnant women, pricing was based on the national health insurance cost at the facility they were attending, which did not guarantee that the testing would be free.

**Table 2:** Median cost of HBV-related care across healthcare facilities in Ghana

	Subsidized price with government health insurance USD Equivalent^a^ Median (Range)	Non-subsidized price without government health insurance USD Equivalent^a^ Median (Range)
*Diagnostics*		
HBsAg	0.89 (0 – 1.94)	2.91 (0.56 – 5.00)
HBV Profile	3.15 (1.96 – 8.07))	7.26 (5.65 – 50.03)
HBV DNA	Not subsidized	64.56 (48.42 – 67.79)
Liver Function	3.23 (0 - 6.46)	9.68 (8.07 – 12.91)
APRI	4.23 (0 – 9.68)	13.71 (2.42 – 16.14)
INR	Not subsidized	8.47 (3.23 – 9.68)
Ultrasound	3.39 (0 – 4.84)	8.88 (6.46 – 19.34)
Abdominal CT	Not subsidized	137.20 (87.16 – 187.22)
Endoscopy	Not subsidized	44.39 (32.28 – 64.56)
Biopsy	Not subsidized	64.56 (48.42 – 80.7)
*Therapeutic*		
Annual cost of tenofovir	Not subsidized	260.50 (145.26 – 290.52)

^a^1 GHS = USD 0.1614 at the time of data collection

### Hepatitis B testing patterns

#### HBsAg rapid diagnostic testing, serological profile and DNA testing

**Fig. 2:**
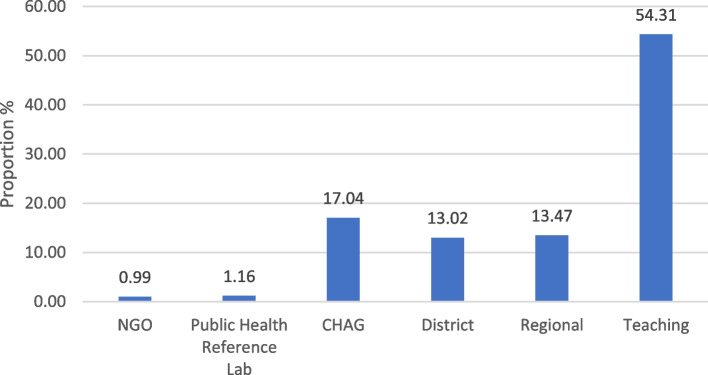
Proportion of HBsAg tests out of total tests done by facility type in 22 health institutions, 2020. Abbreviation: NGO: Non-governmental organisation, CHAG = Christian Health Association of Ghana. NGO (*n* = 2), PHRL (n = 2), CHAG (*n* = 4) District (n = 4) Regional (*n* = 6) Teaching (n = 4)

In 2020, the majority of HBsAg tests were conducted in teaching hospitals, with 26,861 (54.85%) of HBsAg tests performed in these four centres alone (Fig. 2).

**Fig. 3:**
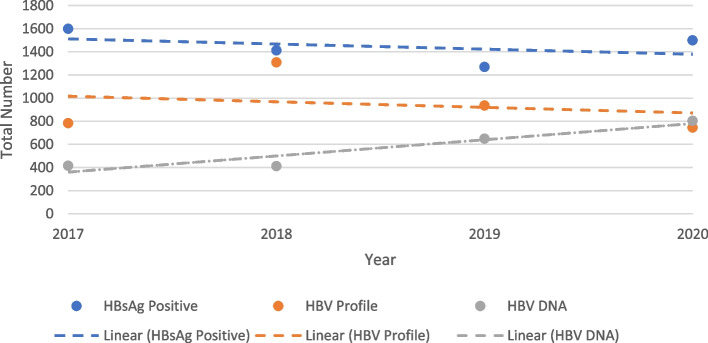
Crude number of HBsAg, HBV serological profile* and HBV DNA tests and their linear trends in two teaching hospitals, 2017-2020 *HBV Serological profile includes Hepatitis B e antigen (HBeAg), Hepatitis B core antibody (HBcAb), Hepatitis B e antibody (HBeAb), Hepatitis B surface antibody (HBsAb) Abbreviations: HBsAg = hepatitis B surface antigen; HBV DNA = hepatitis B virus deoxyribonucleic acid

On review of HBV DNA and serological profile testing data, there was a mismatch between crude number of patients testing HBsAg positive and the total number of HBV serological profile and HBV DNA tests performed with HBV DNA performed to a lesser degree (Fig. 3). However, between 2017 and 2020, there was a close to two-fold increase in the HBV DNA tests performed in the two teaching hospitals. Specifically, in 2017, 1600 HBsAg positive tests were recorded in these facilities, however only 783 serological profile tests and 413 HBV DNA tests were conducted. In 2020, 1500 HBsAg positive tests were recorded in registers, compared with 746 serological profile and 804 HBV DNA tests, and although testing numbers had increased, these two tests still fell short of patients testing HBsAg positive by close to 50%.

### HBV testing in pregnant women

**Fig. 4:**
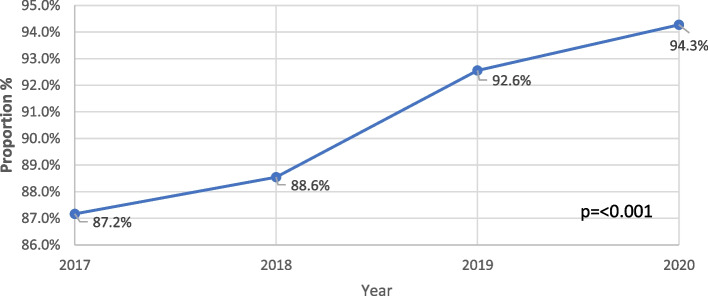
Proportion of pregnant women with hepatitis B surface antigen (HBsAg) test by time of delivery, 2017-2020

**Fig. 5:**
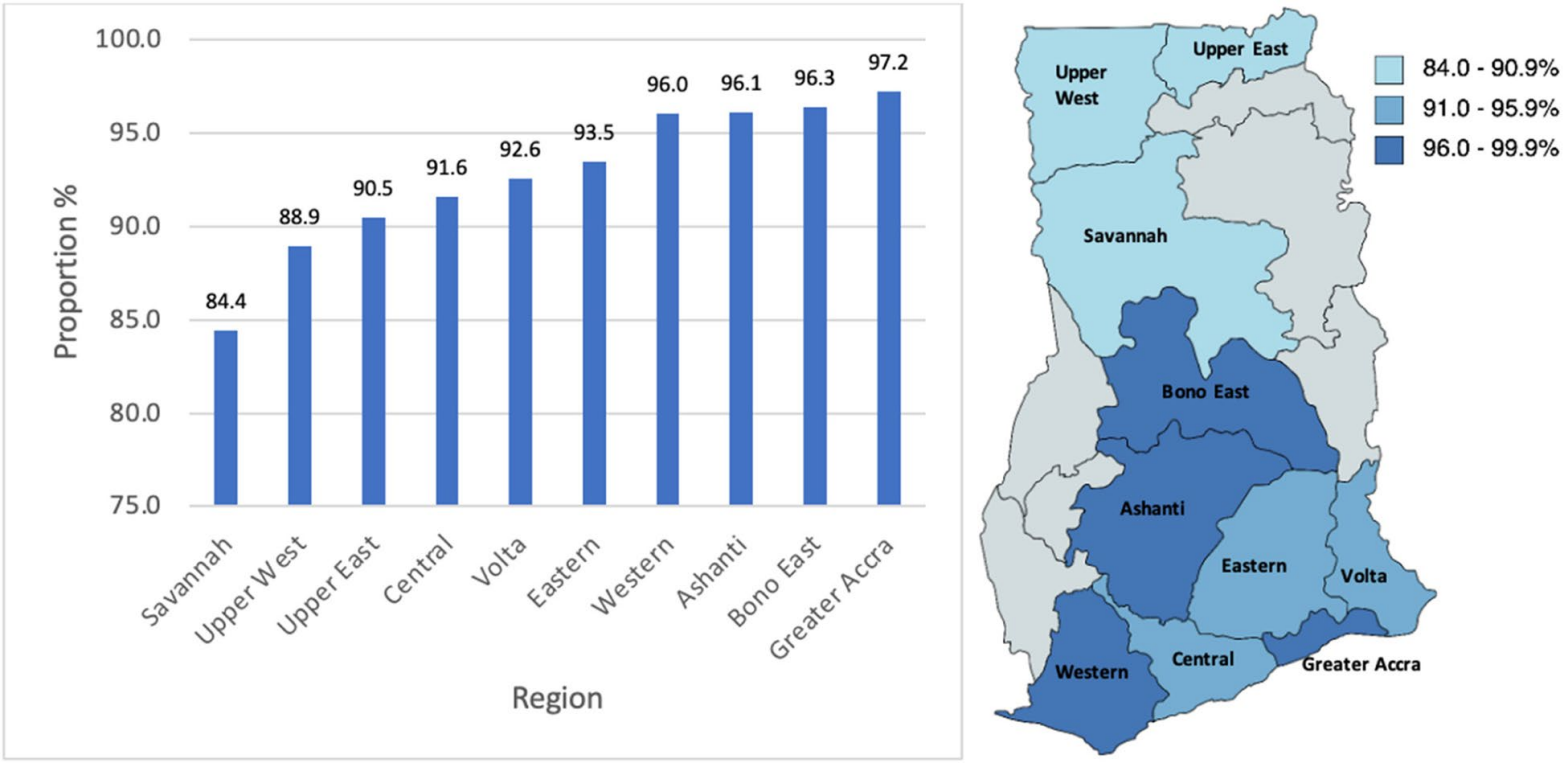
Proportion of pregnant women with hepatitis B surface antigen (HBsAg) test at the time of delivery by region, 2020

The proportion of pregnant women who were tested for HBsAg by the time of delivery increased from 87.2% in 2017 to 94.3% in 2020 (*p* < 0.001) (Fig. 4). Specifically, out of 37,055 parturient women managed on labour wards across 13 healthcare institutions in 2020, 34,933 were tested for HBsAg, whilst 2122 (5.7%) were not, as at the time of delivery. The proportion varied by region, with a lower proportion of parturient women in northern Ghana undertaking HBV testing compared to middle and southern belt regions (Fig. 5). Testing in pregnant women was undertaken during the antenatal clinic visits, usually at the booking (before 16 weeks gestational age) and recorded in the patient’s maternal health record book. At the time of delivery, this information was then captured in the labour ward delivery register.

### HBsAg seroprevalence

#### HBsAg seroprevalence in laboratory-based registers

**Fig. 6:**
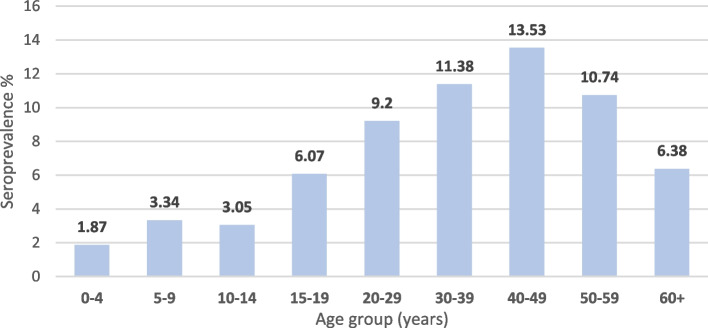
Hepatitis B surface antigen (HBsAg) seroprevalence based on laboratory-based rapid detection tests (RDTs) by age group, 2016–2020. 0-4 years (*n* = 641), 5-9 years (*n* = 539), 10-14 years (*n* = 655) 15-19 years (*n* = 2357) 20-29 years (*n* = 10,133) 30-39 years (*n* = 8556) 40-49 years (*n* = 3741) 50-59 years (*n* = 2160) 60 + years (*n* = 2446)

Crude and pooled estimates for HBsAg seroprevalence were obtained for 12 out of 16 administrative regions using laboratory-based registers. There was limited categorization by disease state, or in- versus out-patient status across institutions among paper-based register records reviewed, except for pregnant women and HIV positive patients. All test results were derived from RDT kits. The median age of persons tested was 31 years (IQR 24-40). Out of 46,542 records where gender was documented, 21,595 (46.40%) of tests were conducted in males and 24,947 (53.60%) in females. A total of 11,874 out of 139,966 persons were HBsAg positive, representing a crude HBsAg seroprevalence of 8.48% (95% CI 8.25 – 8.57%). The pooled estimate, which was a weighted estimate based on the total number of people tested in each region, was 11.40% (95% CI 10.44 – 12.35). The HBsAg seroprevalence among children under 5 years of age (*n* = 641) was 1.87% (95% CI 1.07-3.27) and the highest age-specific seroprevalence was seen in the 40-49 years age-group (Fig. 6).

**Fig. 7:**
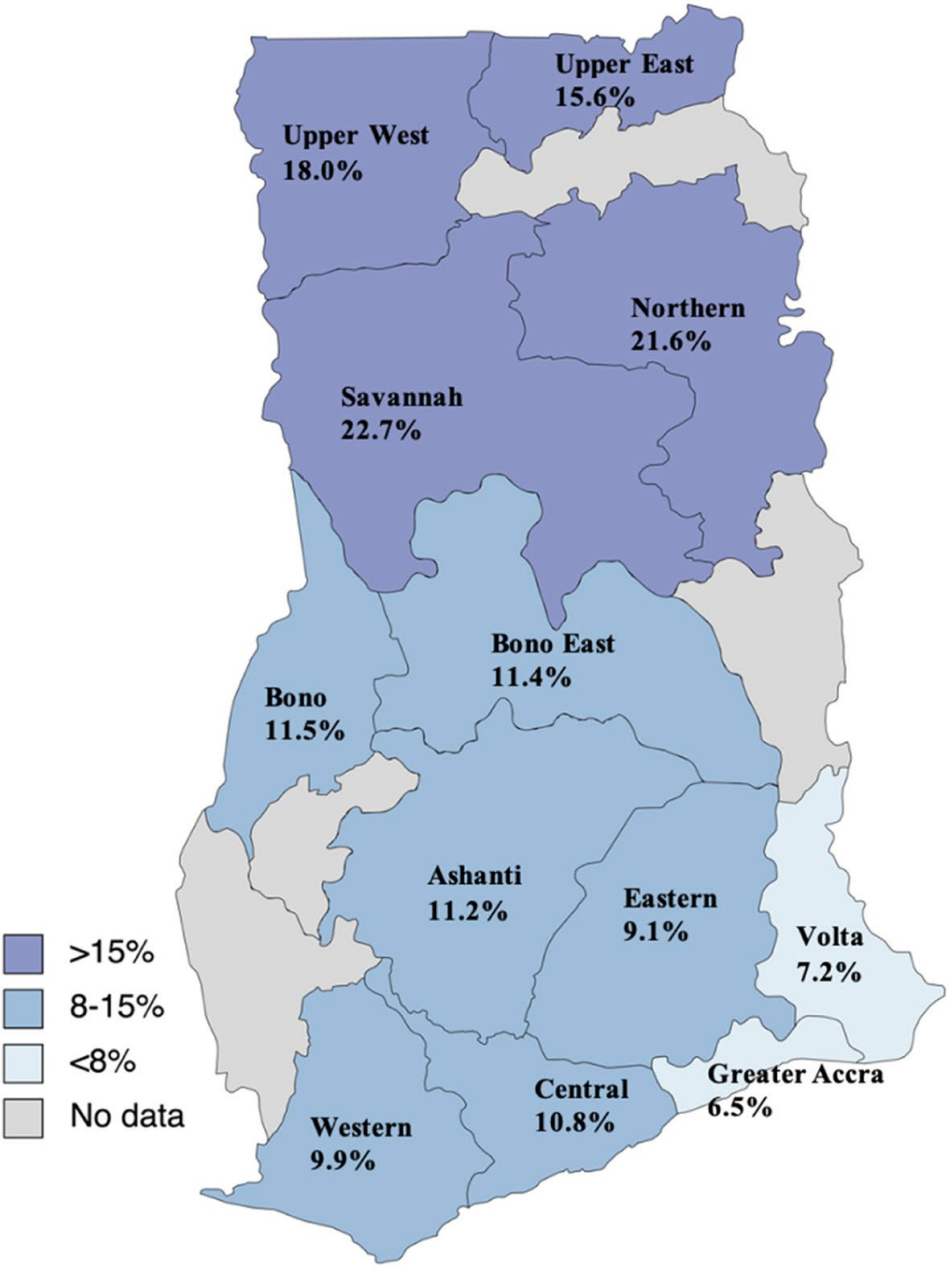
HBsAg seroprevalence based on laboratory-based RDT tests by region, 2016–2020. Map source: https://www.mapchart.net/africa-detailed.html

Age was a predictor of a positive HBsAg RDT test (OR 1.005 95% CI 1.002 – 1.007), and females had lower odds of testing positive (OR 0.81 95% CI 0.74 – 0.88) (Table 3). When the burden across the country was examined, the highest region-specific seroprevalences were in the Savannah (22.7%), Northern (21.6%), Upper West (18.0%), and Upper East (15.6%) regions (Fig. 7).

**Table 3:** Factors associated with hepatitis B surface antigen (HBsAg) seropositivity among hospital attendants between 2016 and 2020

	Adjusted Odds Ratio^a^	95% CI	*p* value
Sex			
Female	0.81	0.74 – 0.88	< 0.001
Age (years)	1.005	1.002 – 1.007	< 0.001
Year			
2016	Ref		
2017	0.69	0.49 – 0.98	0.04
2018	0.66	0.49 – 0.92	0.01
2019	0.77	0.55 – 1.07	0.12
2020	0.68	0.49 – 0.96	0.03
Region			
Volta	Ref		
Central	1.32	1.10 – 1.58	0.003
Eastern	1.15	0.95 – 1.39	0.16
Greater Accra	0.98	0.81 – 1.20	0.87
Bono	1.59	1.27 – 1.98	< 0.001
Upper East	1.93	1.59 – 2.34	< 0.001
Western	1.37	0.86 – 2.20	0.19
Savannah	3.43	2.61 – 4.50	< 0.001

^a^Multivariable model adjusted for age (continuous) gender (male, female), year (categorical) and region (categorical)

### Blood bank registers

**Fig. 8:**
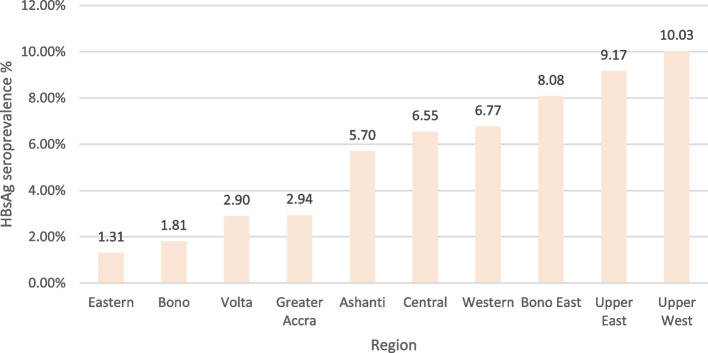
HBsAg seroprevalence among potential blood donors by region based on RDT testing 2016–2020

A total of 9623 out of 169,048 persons tested at blood banks were HBsAg positive, representing a crude seroprevalence of 5.69% (95% CI 5.58 – 5.80). The pooled estimate among potential blood donors was 5.12% (95% CI 4.26-5.98). The highest regional burden was seen in Upper West (10.03%) and Upper East Regions (9.17%) (Fig. 8), and these findings were consistent with burden estimates obtained from laboratory registers.

### Delivery ward registers

**Fig. 9:**
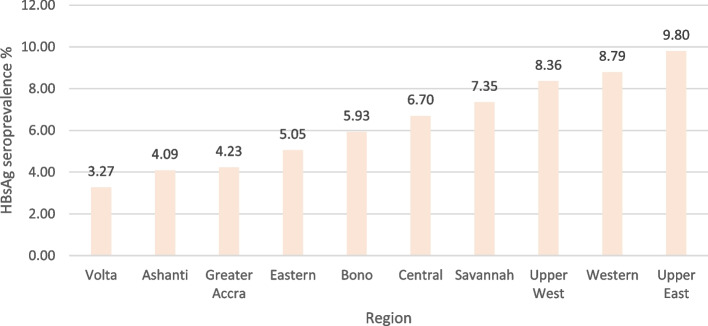
HBsAg seroprevalence among pregnant women by region in Ghana 2017 – 2020

The crude HBsAg seroprevalence among pregnant women (*n* = 83,920) was 6.14% (95% CI 5.97-6.31). The pooled estimate was 6.36% (95% CI 5.70-7.02). Seroprevalence was highest in the 20-29 years (6.18%) and 30-39 years (7.01%) age groups. Figure 9 demonstrates the region-specific seroprevalence amongst pregnant women. The highest burden was in the Upper East Region (9.80%), whilst the lowest was in the Volta region (3.27%).

### Prison inmates

Data was obtained for 274 inmates who were tested for HBsAg as part of an NGO health screening exercise among prisoners in the Central Region of Ghana in 2019. The HBsAg seroprevalence among prison inmates was 13.14% (Table 4). The seroprevalence was significantly higher in male (18.76%) than female inmates (3.06%) *p* < 0.001.

**Table 4:** HBsAg seroprevalence by sex and HIV status among prison inmates and PLHIV

	HBsAg positive n/N (%)	*p* value
Prison inmates^a^ (both sexes)	36 / 274 (13.14)	
Male	33 / 176 (18.75)	< 0.001
Female	3 / 98 (3.06)	
HIV Status		
HIV+	21 / 344 (6.10)	0.349
HIV-	538 / 9837 (5.47)	

Data source for prison inmates: Hepatitis Foundation of Ghana. Data source for HIV Status: Blood bank registers

^a^Data obtained from one male prison and one female prison in Central and Eastern regions respectively. *Abbreviations:* PLHIV = persons living with human immunodeficiency virus

### Persons living with HIV

The HBsAg test results for patients who tested positive for HIV infection (*n* = 344) across multiple centres were reviewed. The HBsAg seroprevalence among persons living with HIV (PLHIV) was 6.10% (Table 4).

### HBV serological profile

Although all study sites provided records on RDT testing for HBsAg, only three sites provided register entries for HBV serological profile including HBcAb and HBeAg. The results of 1268 HBV serological profile results based on RDT and ELISA tests conducted between 2018 and 2021 were therefore reviewed (Table 5). Of those tested, 7.32% had evidence of immunity as demonstrated by a positive Hepatitis B surface antibody (HBsAb) result. An estimated 6.28% (95% CI 4.73 – 7.84) of patients with chronic HBV infection were e antigen positive (Table 6), with males having higher e-antigen seropositivity (OR = 1.62, 95% CI: 0.98 – 2.60), though this difference was not statistically significant (*p* = 0.058). The proportion of individuals with resolved infection after natural exposure (negative HBsAg with positive HBcAb) was 0.7% (95% CI 0.2-1.2). HBcAb positivity was associated with age greater than 14 years e.g., 15-19 years OR = 9.17 95% CI: 1.72 – 48.94; 20-29 years OR = 9.67 95% CI 2.23 – 42.05, etc. compared to the baseline group of 0–4-year-olds (Table 7).

**Table 5:** Results of patients undergoing HBV serological profile testing (*n* = 1268)

	Positive n, %	Negative n, %
HBsAg	992 (78.48)	272 (21.52)
HBeAg	68 (5.41)	1188 (95.59)
HBcAb	1002 (79.59)	257 (20.41)
HBeAb	629 (49.96)	630 (50.04)
HBsAb	92 (7.32)	1164 (92.68)

*Abbreviations: HBsAg* Hepatitis B surface antigen, *HBeAg* Hepatitis B envelope antigen, *HBcAb* Hepatitis B core antibody, *HBeAb* Hepatitis B envelope antibody, *HBsAb* Hepatitis B surface antibody

**Table 6:** E antigen status of chronic HBV cases (2018-2021)

	Chronic HBV infection n (%)	Hepatitis B e antigen (HBeAg) positive chronic HBV n (%)	Odds ratio (95% CI)
All patients	924 (97.88)	58 **(6.28)**	
Female	459 (99.35)	25 (5.45)	Ref
Male	377 (97.67)	32 (8.49)	1.62 (0.98-2.60)

Data on sex available for 836/924 records

**Table 7:** Anti-HBc positive test result by age-group

Age Group (years)	Crude OR	95% CI	*p* value
0-4	Ref		
5-9	0.33	0.03 - 4.40	0.40
10-14	0.95	0.144 - 6.28	0.96
15-19	9.17	1.72 - 48.94	**0.01**
20-29	9.67	2.23 - 42.05	**0.002**
30-39	10.86	2.49 - 47.34	**0.002**
40-49	7.5	1.69 - 33.24	**0.008**
50-59	6.04	1.30 - 28.03	**0.022**

## Discussion

In our evaluation, although registers did not capture reasons for conducting HBV testing, facility personnel reported the primary reason for testing was risk-based or provider-initiated assessment, which is typical of facility-based testing. NGOs included in the study conducted HBsAg testing by population-based screening periodically, depending on funds available, and these were generally considered insufficient in frequency and coverage to identify a significant number of the populace. Studies assessing testing practices in low- and lower middle-income countries found that in general, HBV testing is doctor-initiated, with only few countries in Africa stating that there is a population- or community-based screening programme in place [16, 17]. In Ghana, HBV testing is risk-based [18], with no current national or population-based screening programme in place. This method of testing includes screening based on clinical presentation or risk behaviours such as injection drug use. Consequently, estimates derived from risk-based testing are biased and higher than estimates from a random serosurvey. Population-based screening in regions with HBV prevalence of > 2% is recommended by the WHO, especially among pregnant women [19]. Since Ghana has high endemicity for HBV infection, it is imperative that such an agenda is put into action, with subsequent linkage to care for those testing positive.

In the present study, the proportion of pregnant women who had undertaken HBV screening as at the time of delivery was also determined using labour ward registers. We found that for the year 2020, 94.3% of pregnant women delivering in health facilities had received an HBsAg test, and that the rate of testing among pregnant women had steadily increased between 2017 and 2020. Among pregnant women in Ghana, HBsAg testing is required as part of routine testing for antenatal care registration. Mother-to-child transmission is a major route of HBV transmission in low- and middle-income countries. To achieve elimination goals for HBV transmission, WHO recommends all infants receive hepatitis B birth dose vaccination, to screen all pregnant women for HBsAg and if positive to deliver HBIG if available to their infants. Additionally, it is recommended to deliver appropriate maternal antiviral prophylaxis by screening for evidence of high viral load of HBV based on HBV DNA or if not available HBeAg testing [20]. Some studies have suggested that the rate of HBV screening among pregnant women in regions of high endemicity are low [21, 22], therefore the high and increasing proportion of pregnant women tested in Ghana since 2017 is promising. However, it must be noted that our data were obtained from health facilities. Women delivering outside of health facilities may have different health-seeking behaviours and different access to prenatal care and HBV testing rates. Further research is needed to determine the reasons for various health seeking behaviours, to establish barriers to antenatal care and delivery in health centres, to determine the current proportion of women giving birth outside healthcare centres, and to determine the rates of HBV and MTCT in these groups.

Also, data are lacking regarding the proportion of HBsAg+ women who were linked to care for their health, or who received further testing for HBeAg or HBV DNA. A pilot study conducted in the Eastern Region of Ghana found that only 6 and 1% of women received HbeAg and HBV DNA testing, respectively following an HbsAg positive test [23]. To benefit from HBV testing, it is therefore imperative that the promising screening rates are complimented by comparable rates of serological and HBV DNA testing, and linkage to care, if PMTCT of HBV is to be successful. Furthermore, data are needed in Ghana regarding the proportion of HBV-exposed babies who receive appropriate care (HBV birth dose vaccination and HBIG), as well as outcomes of infants born to HbsAg+ mothers who receive these interventions.

A positive HBsAg result is followed by serological profile testing, HBV DNA testing, and assessments of hepatic structure and function to determine whether treatment criteria are met [24]. In the present study, we noted a significant mismatch between HBsAg+ results and HBV serological profile and DNA testing. Limitations in testing for HBV serological profile and HBV DNA in LMICs are often a result of cost of assays and lack of government financing of HBV testing, lack of or non-functioning equipment outside of large referral centres and delays caused by centralized laboratory systems [25, 26]. Such limitations make it difficult to determine which patients meet treatment criteria, and the opportunity to initiate treatment or limit transmission is potentially lost. Conversely, patients who do not require treatment may be initiated on therapy without enough laboratory information. We found that HBV DNA testing was limited to the largest teaching hospitals. Decentralisation of HBV testing, and creation of simple and understandable algorithms for care in LMICs can expand HBV testing and treatment to meet elimination targets. To elaborate, current orthodox algorithms require use of HBV DNA level cut offs based on Hepatitis B e Antigen status, however, simpler algorithms such as TREAT-B which does not require HBV DNA, have been demonstrated to be less costly and applicable in low-resource settings for determining treatment eligibility [27–29]. Another proposed method is to link HBV care to already established national HIV and tuberculosis (TB) control programmes. For example in the national TB programme, PCR testing sites are widely available across Ghana [30] in the form of GeneXpert® systems. Exploring the use of these systems under dialogue and collaboration with government and funding agencies, may be one way of increasing PCR testing capacity for HBV.

In our study, the crude and pooled HBsAg seroprevalence estimates excluding blood donors and pregnant women were 8.4 and 11.4% respectively. Abesig et al. in their systematic review of studies conducted between 2015 and 2019, found a national prevalence in the adult population of 8.4% with lower prevalence in the northern part of Ghana (5.7%) compared to the south (8.9%) [12].

Conversely, we found higher region-specific seroprevalence in the Northern regions of Ghana, specifically in the Upper East (15.6%), Upper West (18.0%), Savannah (22.7%) and Northern regions (21.6%). This higher region-specific prevalence in our study, compared to the recent systematic review by Abesig et al. could be because of the differences in the study population between the two studies. In their paper, estimates from the Northern regions were derived from studies in pregnant women, whilst studies in southern Ghana included outpatients, patients with HIV and patients with jaundice. Our study largely comprised data hospital attendants and some community-based screening. There may be multiple factors that contribute to the higher HBV seroprevalence in Northern Ghana found in our study. Disparities in the level of health care compared with the south, challenges in implementing practices promoting maternal and child health, insufficient healthcare staff, lower educational level, socio-cultural factors, and higher poverty rates [31] are likely to contribute to the increased burden of HBV in these areas. Eliminating HBV in Ghana needs focused policies that will address these gaps in healthcare in Northern Ghana, including increasing testing and treatment capacity, and strategies to prevent mother-to-child-transmission.

In the present study, we found that the HBsAg seroprevalence in children < 5 years was 1.9%. Modelled estimates of the global prevalence in children < 5 years range between 1.3 - 3.4%, and it is estimated that the HBsAg seroprevalence in Ghana for children < 5 years is between 0.9 - 1.4% [2]. This indicator is important, as it can be used to measure the success of Hepatitis B vaccination programmes, as well as for estimation of the cumulative incidence of chronic HBV infection [2]. Absolute elimination targets include a seroprevalence of < 0.1% in children under 5 years by 2030 [32], therefore our estimate suggest that Ghana has not yet reached this target and has just 8 years to do so.

We found that the prevalence among adolescents (15 – 19 year olds) was 6.9%, which was much lower than a previously reported estimate of 14.3% in 2016 [12]. This is important, because Hepatitis B vaccination at 6, 10 and 14 weeks of age was introduced into the expanded program for immunisation (EPI) in Ghana in 2002. Therefore, the early recipients of this program are in the 15-19 years age group of this current study. Consequently, it is interesting to compare HBsAg seroprevalence among adolescents in our study with that of the 2016 study, since it may reflect a significant decline in HBsAg seroprevalence in a vaccinated cohort. A pilot study conducted in Ghana, supports this finding, in that the HBcAb seroprevalence among school children was higher (6.1%) in children born before the introduction of childhood HBV vaccination, compared with after (2.6%) its introduction [33]. Furthermore, when the yearly coverage for HBV vaccination among 1-year olds in Ghana in compared over the past two decades, it is noted that in 2002, when the EPI began in Ghana, coverage was 80% compared with 94% in 2012 and 98% in 2022 [34]. This is important to reflect that increasing vaccine coverage in Ghana has likely contributed to the decreasing prevalence among age-groups below 20 years, as seen in our data.

In our study, the HBsAg seroprevalence among pregnant women (6.4%) and blood donors (5.0%) was lower than previously reported pooled estimates of 7.4 -13.1% and 7.2 - 11.6% respectively from systematic reviews [12, 13]. This may suggest a decline in HBV burden, since studies used to derive these pooled estimates were conducted between 1995 and 2017, compared with our data which spanned 2016 – 2021. Furthermore, on multivariable analysis, our findings suggested that the odds of testing HBsAg positive were significantly lower for each year between 2017 and 2020, compared with 2016.

The HIV-HBV seroprevalence of 6.1% in this study is lower than a previously reported estimate for SSA [35]. In Ghana, two studies reported the co-prevalence as 6.1 and 12.3% in the Central and Ashanti regions respectively [36, 37]. Screening for HBV in PLHIV is important because there is a higher risk drug related liver toxicity as well as HCC development in these patients [38] Furthermore, screening is necessary for appropriate drug selection in HIV treatment, and to identify HBV negative individuals, for whom HBV vaccination must be offered to reduce the chances of infection.

The strengths of this study include the wide coverage of administrative regions and facility type from where data was obtained, which allowed some comparisons between regions and helped to obtain a potentially nationally representative picture of the patterns of HBV testing in Ghana. It is however noted that sites were selected using non-probability sampling, and that precise comparisons were limited due to the nature of data available. Nonetheless, for the first time, we were able to determine seroprevalence in regions such as the Upper West and Savannah regions, which were not previously available. Furthermore, we provided an updated estimate of seroprevalence in children < 5 years.

Our study was not without limitations. We used secondary data for this assessment, therefore there was some missing data. Additionally, some data, such as the proportion of HBV exposed babies who receive appropriate PMTCT were unavailable because they were not routinely recorded. Furthermore, for regional data, it is possible that a few patients visited health facilities outside their region of residence, therefore this may have had minimal impact on the regional prevalence. Limitations notwithstanding, we believe the large dataset obtained allows for reliable analysis and our subsequent estimates. We also note that a higher pooled seroprevalence from our study may be because data was obtained from hospital laboratory registers. However most previous studies, including those used in the systematic reviews were similarly based on hospital-level data, with few community or population-based studies. We therefore believe our data is comparable to these.

## Conclusion and recommendations

In conclusion, Ghana remains a country with a high burden of hepatitis B virus infection. Northern regions demonstrate a higher HBsAg seroprevalence, and a relatively lower proportion of pregnant women undertake HBsAg testing before delivery, compared with regions in middle and southern Ghana. Furthermore, PCR testing is not widely available outside of large teaching hospitals, which limits diagnostic work-up, and there is no subsidy for this test, nor antiviral medication in the National Health Insurance scheme, with patients still currently paying out-of-pocket. As a highly endemic country for HBV, increased testing and treatment of HBV remains a key strategy in overcoming the burden of HBV and its sequelae. Consideration of population-based screening methods may be helpful if systems are put in place to ensure linkage to care. Where gains have been made in testing, for example among pregnant women, this must be complimented with follow through testing for serological profile and HBV DNA, linkage to appropriate care, and PMTCT practices such as birth-dose vaccination. This remains a key part of reducing HBV transmission, as well as preventing the long-term sequelae of chronic HBV infection, such as liver cirrhosis and hepatocellular carcinoma. The foreseeable challenges with implementing these strategies in an LMIC such as Ghana includes funding gaps, therefore a funding plan for elimination is important and necessary to allow planning for appropriate budgetary allocations. Government agencies must be encouraged and political will secured, if LMICS such as Ghana are to be able to undertake such programmes to overcome the burden of disease. Testing sites must move beyond HBV RDT testing alone, and access to serological profile testing and PCR must be expanded especially to Northern Ghana, where burden of disease is highest, so that treatment decisions can be readily made. Furthermore, hepatitis reporting systems and registers should be firstly improved to facilitate data capture of important elimination indicators and standardised across the country to allow for comparability.

## Data Availability

The datasets used and/or analysed during the current study are available from the corresponding author on reasonable request.

## Abbreviations

ANC: Antenatal clinic
CHAG: Christian Health Association of Ghana
ELISA: Enzyme linked immunosorbent assay
EPI: Expanded program for immunisation
HBV: Hepatitis B
HBcAb: Hepatitis B core antibody
HBsAg: Hepatitis B surface antigen
HBeAg: Hepatitis B envelope antigen
HCC: Hepatocellular carcinoma
HIV: Human Immunodeficiency Virus
LMIC: Low and middle-income countries
NGO: Non- Governmental Organisation
PLHIV: Person living with HIV
PMTCT: Prevention of mother to child transmission
RDT: Rapid diagnostic test
SSA: Sub-Saharan Africa
WHO: World Health Organisation
